# Analysis of the Relationship Between Organizational Learning and Healthcare Integration

**DOI:** 10.5334/ijic.9681

**Published:** 2025-05-09

**Authors:** Roberto Nuño-Solinís

**Affiliations:** 1 Matia Institute, and Lecturer in Healthcare Management, UNIR, Spain

**Keywords:** integrated care, organizational learning, chronic conditions, evaluation, positive deviants, leadership

## Abstract

This thesis explores the relationship between organizational learning and integrated care in health systems. Through a mixed-methods approach, it analyzes how healthcare organizations develop learning capabilities and how these contribute to the effectiveness of integrated care models. These findings indicate that fostering a culture of continuous learning enhances care coordination and patient outcomes. However, structural and cultural barriers may hinder learning processes. This study provides insights for policymakers and healthcare managers on leveraging organizational learning to improve integrated care implementation.

## Background

Integrated care is a central component of healthcare transformation agendas worldwide, in response to the existing challenges of health systems. These challenges include demographic and epidemiological transitions (aging, chronicity and multimorbidity), as well as the uptake of innovation, while ensuring quality, efficiency, and sustainability.

In Spain, numerous initiatives under the “integrated care” umbrella have been implemented, but most remain unevaluated [1], contributing to the so-called organizational “black box” [2]—a lack of understanding of the organizational dynamics that drive integration success. This thesis applies organizational learning theories to illuminate this black box and establish a pioneering link between the fields of integrated care and organizational learning, considering that previous research in this field has been limited and scarcely systematized [3,4,5].

## Research Questions

This thesis arises from an interest in investigating and deepening the process by which health organizations learn and how they improve their performance as a result of this learning and how their integrated care advancement influences these dynamics.

The ultimate goal is to identify teachings from organizational learning theory that can be useful in understanding the dynamics of care integration in the Spanish National Health System in a context in which this paradigm is being established from an academic, political-strategic, and care delivery perspective.

## Methods

A mixed-method design was employed, combining quantitative and qualitative research approaches. This study developed an Analytical Model to explore the relationship between organizational learning capacity, integrated care, and organizational performance. This model was applied to eight Spanish healthcare organizations recognized for excellence in integrated care [6] (positive deviants), measured through avoidable hospitalizations for chronic conditions. This research adopted a qualitative, exploratory, and comparative case study approach.

## Main findings and contributions

- From a review of the state of the art, a conceptual connection between organizational learning and healthcare integration was established. It was shown that healthcare organizations could improve their results in terms of the Quadruple Aim [7] by improving their organizational learning capacity [8].
- As a result of the development of the analytical model, a taxonomy of high-performing healthcare organizations in Spain was established:
  - Historically Integrated Health Organizations. Vertically integrated organizations that have consolidated integration cultures, through three decades of evolution (e.g., SSIBE: Baix Empordá Integrated Health Services).
  - Accountable Care Model. Based on the US-inspired orthodoxy of accountable care, adapting a population health management model publicly funded by a per capita contract and full managerial autonomy of the commissioned provider (e.g., Marina Salud).
  - Non-integrated Business Model. Non vertically integrated Hospitals that have legal personality, strong managerial autonomy that allows innovative alliances with primary care (e.g., Aljarafe Healthcare Consortium, Costa del Sol Hospital).
  - Second-generation Integrated Healthcare Organizations. Publicly managed and vertically integrated organizations without legal personality that have been developed in recognition of the need of care integration to provide better care for chronic patients (e.g., Bidasoa Integrated Health Organization, Serranía de Málaga Health Area).
- Organizational learning features contribute to the advancement of integrated care, particularly clinical governance was the backbone that connects organizational practices linked to learning and organizational actions that favor integration. The presence of shared objectives between care levels with regular monitoring and systematic feedback to staff was a decisive driver. Aligned leadership between primary care and hospital settings, managerial stability and the presence of primary care executives in top managerial structures facilitate care integration initiatives.
- Healthcare organizations with strong learning cultures exhibit higher levels of care coordination and improved patient outcomes. The existence of a learning support environment is closely linked to the patient safety culture and the deployment of continuous improvement methods. Top-performing organizations show long-term commitment and managerial practices aligned with reference models such as EFQM or the Joint Commission. Moreover, organizations that invest in knowledge-sharing mechanisms such as training programs and reflective practices enhance their integrated care effectiveness.
- The findings of the comparative study among top performers show a positive relationship between learning capacity and care integration in all analyzed dimensions. Similarly, a virtuous cycle can be observed between integrated care features (normative or functional) as drivers of the learning process. This interrelation is highly complex, and it is not possible to identify a single pattern. Therefore, it can be stated that each organization is building its own path of organizational learning modeled and modulated by its environment and organizational characteristics. Some common organizational learning patterns were identified.
  - Structured workforce training programs linked to broader organizational strategies facilitate continuous learning.
  - Innovative tools and interventions encourage knowledge exchange and contribute to care integration, particularly comprehensive programs focused on patients with multimorbidity and population stratification models.
  - Benchmarking is a common practice among top performers, particularly in a subset of organizations under greater financial pressure.
  - Advanced information systems and decision support tools play a crucial role in consolidating organizational memory.

## Implications for Integrated Care and Future Research

The findings underscore the importance of fostering learning-oriented healthcare organizations in strengthening integrated care. Moreover, the transformation towards more integration of care could be conceptualized as an organizational learning journey [9]; this relationship is complex, systemic and multilevel and therefore the lens of organizational learning helps to understand service integration and reflect on some of the challenges faced by organizations in developing a learning culture [10].

Policymakers should prioritize strategies that encourage continuous professional development, interdisciplinary collaboration, and organizational adaptability. Future research should explore the longitudinal impact of learning interventions on integrated care outcomes and identify best practices for embedding learning in healthcare systems.

From a policy and healthcare management perspective, the key recommendations are as follows:

- Recognizing integrated care as a strategic driver of healthcare sustainability, with organizational learning as a critical enabler.
- Scaling up integrated care innovations that promote learning, such as patient-centered models for multimorbid individuals, integrated care pathways, and population risk stratification systems.
- Expanding vertical integration strategies to enhance shared learning between care levels.
- Ensuring managerial stability, as high turnover hinders organizational learning.
- Aligning clinical and managerial leadership, providing healthcare organizations with decision-making autonomy, and fostering a long-term vision for transformation.

We hope that the insights from this doctoral thesis will contribute to informed decision-making and ultimately benefit healthcare systems by fostering more integrated and learning-oriented organizations.

## Author information

Roberto Nuño-Solinís is a researcher in the field of Organizational Theory in healthcare with a main focus on integrated care. His doctoral thesis entitled “Analysis of the Relationship between Organizational Learning and Integrated Care in Healthcare” [11] was defended on September 30, 2020, at the Department of Economics and Business Administration of the Universidad a Distancia de Madrid under the supervision of Professor Eduardo Bueno Campos. The thesis received the highest distinction (Outstanding Cum Laude), with a tribunal composed of Professors Gregorio Martín de Castro (President, Universidad Complutense de Madrid), Pedro Aceituno-Aceituno (Secretary, Universidad a Distancia de Madrid), and Mª Paz Salmador Sánchez (Vocal, Universidad Autónoma de Madrid).
